# Supplementary material for: The interactome of intact mitochondria by cross-linking mass spectrometry provides evidence for coexisting respiratory supercomplexes
Source: Mol Cell Proteomics. 2017 Dec 8;17(2):216–32. doi: 10.1074/mcp.RA117.000470 (PMC5795388; doi:10.1074/mcp.RA117.000470)
Supplement: Supplemental Data [file supp_RA117.000470_133922_0_supp_23978_fzffwf.zip › spectra_annotation/mito_DR_spectra_annotation/421-1-4-1-18-1.pdf]

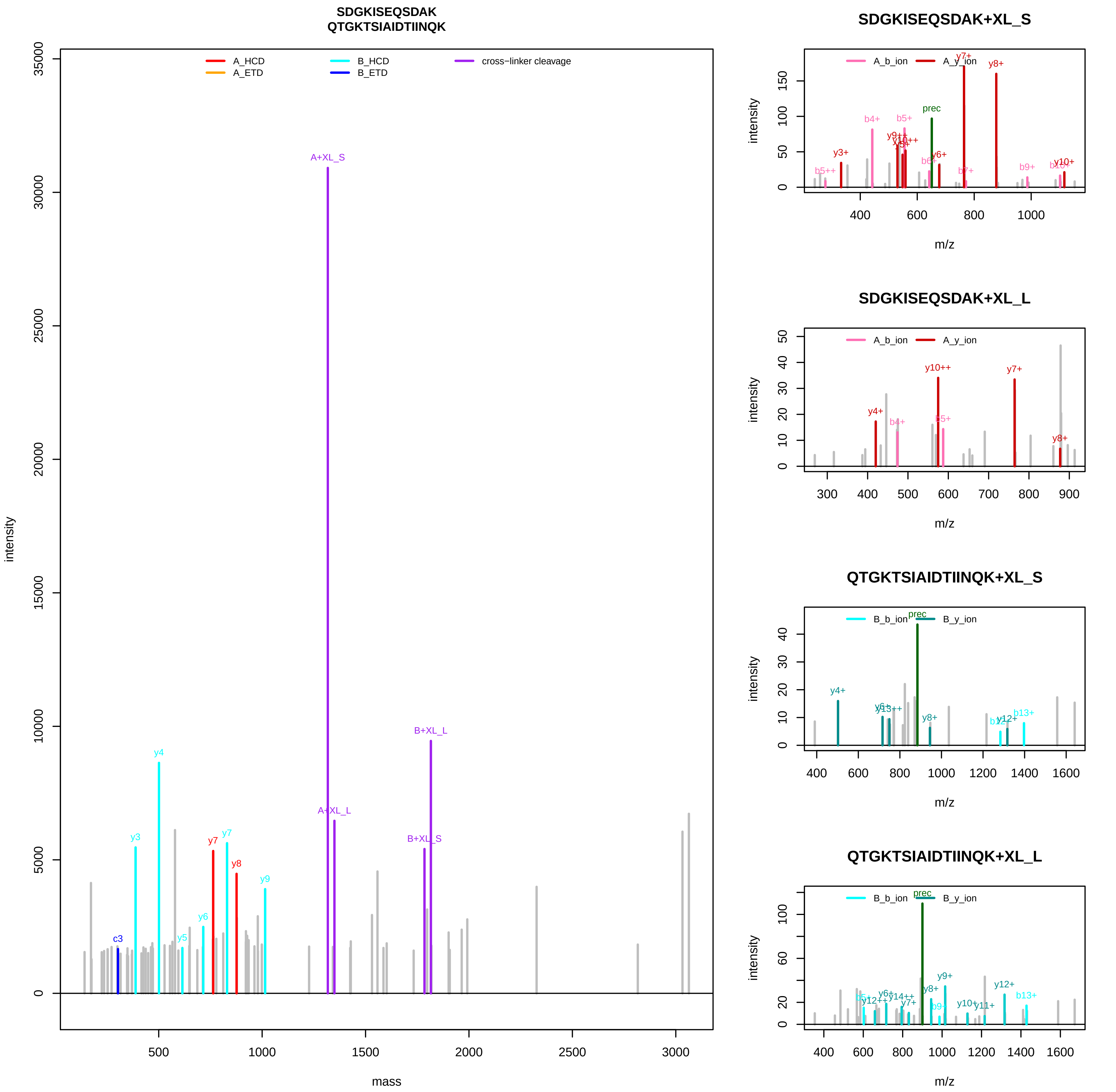

SDGKISEQSDAK
QTGKTSIAIDTIINQK
A_HCD
A_ETD
B_HCD
B_ETD
cross−linker cleavage
A+XL_S
A+XL_L
B+XL_L
B+XL_S
c3
y3
y4
y5
y6
y7
y8
y9
intensity
mass
0
5000
10000
15000
20000
25000
30000
35000
500
1000
1500
2000
2500
3000
SDGKISEQSDAK+XL_S
A_b_ion
A_y_ion
prec
b4+
b5+
b5++
b6+
b7+
b9+
b10+
y3+
y6+
y7+
y8+
y9++
y10++
y10+
intensity
m/z
SDGKISEQSDAK+XL_L
y4+
b4+
b5+
y7+
y8+
y10++
QTGKTSIAIDTIINQK+XL_S
B_b_ion
B_y_ion
y4+
y6+
y8+
y12+
b13+
y13++
QTGKTSIAIDTIINQK+XL_L
y6+
y7+
y8+
y9+
y10+
y11+
y12+
y12++
y14++
b13+
b9+
b5+
